# In vitro and in vivo cell invasion and systemic spreading of *Mycoplasma agalactiae* in the sheep infection model

**DOI:** 10.1016/j.ijmm.2014.07.011

**Published:** 2014-11

**Authors:** Shivanand Hegde, Shrilakshmi Hegde, Joachim Spergser, René Brunthaler, Renate Rosengarten, Rohini Chopra-Dewasthaly

**Affiliations:** a Division of Clinical Microbiology and Infection Biology, Institute of Bacteriology, Mycology and Hygiene, Department of Pathobiology, University of Veterinary Medicine, Veterinaerplatz 1, A-1210 Vienna, Austria; b Institute of Pathology and Forensic Veterinary Medicine, Department of Pathobiology; University of Veterinary Medicine, Veterinaerplatz 1, A-1210 Vienna, Austria

**Keywords:** *Mycoplasma agalactiae*, Cell invasion, Systemic spreading, Persistence, Immunohistochemistry, Intracellular

## Abstract

Generally regarded as extracellular pathogens, molecular mechanisms of mycoplasma persistence, chronicity and disease spread are largely unknown. *Mycoplasma agalactiae*, an economically important pathogen of small ruminants, causes chronic infections that are difficult to eradicate. Animals continue to shed the agent for several months and even years after the initial infection, in spite of long antibiotic treatment. However, little is known about the strategies that *M. agalactiae* employs to survive and spread within an immunocompetent host to cause chronic disease. Here, we demonstrate for the first time its ability to invade cultured human (HeLa) and ruminant (BEND and BLF) host cells. Presence of intracellular mycoplasmas is clearly substantiated using differential immunofluorescence technique and quantitative gentamicin invasion assays. Internalized *M. agalactiae* could survive and exit the cells in a viable state to repopulate the extracellular environment after complete removal of extracellular bacteria with gentamicin. Furthermore, an experimental sheep intramammary infection was carried out to evaluate its systemic spread to organs and host niches distant from the site of initial infection. Positive results obtained via PCR, culture and immunohistochemistry, especially the latter depicting the presence of *M. agalactiae* in the cytoplasm of mammary duct epithelium and macrophages, clearly provide the first formal proof of *M. agalactiae's* capability to translocate across the mammary epithelium and systemically disseminate to distant inner organs. Altogether, the findings of these in vitro and in vivo studies indicate that *M. agalactiae* is capable of entering host cells and this might be the strategy that it employs at a population level to ward off the host immune response and antibiotic action, and to disseminate to new and safer niches to later egress and once again proliferate upon the return of favorable conditions to cause persistent chronic infections.

## Introduction

Commonly described as the smallest and simplest bacteria, mycoplasmas are important pathogens of humans and animals with rather complex and sophisticated pathogenic attributes (Rosengarten et al., 2000). Having lost many of their metabolic pathways during a so-called degenerative evolution from a Gram-positive ancestor, these wall-less prokaryotes readily obtain their nutrition from host cells by mostly colonizing epithelial surfaces and thereby cause slow-progressing chronic diseases that are difficult to cure (Razin et al., 1998). Well-known for their antigenic variation systems, they have adapted sophisticated mechanisms to evade immune clearance, survive in the host and have evolved to infect new host niches (Rottem and Barile, 1993; Chopra-Dewasthaly et al., 2012; Citti and Blanchard, 2013). Mycoplasmas lack typical bacterial virulence factors like toxins, and the molecular determinants of their pathogenicity are largely unknown. This can be partially attributed to their fastidious and slow growth, relative recalcitrance to genetic manipulations, and also to their strict host-specificity that hinders the development of convenient small animal models (Razin et al., 1998; Citti and Blanchard, 2013).

*Mycoplasma agalactiae* is an economically important pathogen and the main etiological agent of contagious agalactia (CA) syndrome in sheep and goats, mainly characterized by mastitis, conjunctivitis and arthritis as predominant symptoms of a localized infection. Sporadically septicaemia, arthritis, pneumonia and reproductive disorders have also been reported (Bergonier et al., 1997; Gomez-Martin et al., 2013), indicating that the pathogen is capable of crossing the epithelial barrier to reach distant host niches, as also evidenced in a recent report where naturally infected asymptomatic male goats were shown to harbour *M. agalactiae* in atypical inner organs like brain and heart (Gomez-Martin et al., 2012). Nevertheless, *M. agalactiae* has so far been regarded as an extracellular parasite and it is unknown how it transforms local infections into systemic ones.

Persistence and chronicity are other hallmarks of *M. agalactiae* infections. Both diseased and asymptomatic animals continue to shed the pathogen for long periods of time, sometimes lasting up to several years (Bergonier et al., 1997). Antibiotic treatments are often unsuccessful as they can only reduce clinical symptoms but tend to promote the carriers that stay unaffected (Nicholas, 2002). Chronically infected and serologically negative herds with no signs of disease are a common clinical-epidemiological situation in endemic areas. Such animals easily escape disease control and eradication measures, and are capable of flaring up frequent CA outbreaks under stress conditions (Gomez-Martin et al., 2013) leading to huge economic losses. Despite such agronomical significance*, M. agalactiae's* pathogenic determinants and mechanisms of infection and persistence are largely unknown, a fact that can be attributed to its long resistance to genetic manipulation until 2005 (Chopra-Dewasthaly et al., 2005a, b), and also because it does not exhibit the more practical phenotypes associated with mycoplasma pathogenicity, such as hemadsorption and the presence of terminal tip structure as attachment organelle, and lacks convenient small animal models or cell lines for appropriate studies.

*M. agalactiae* demonstrates surface antigenic diversity via high-frequency switching of six immunodominant surface lipoproteins (Vpmas) caused by Xer1 recombinase encoded on the same pathogenicity island-like locus (Glew et al., 2002; Chopra-Dewasthaly et al., 2008; Czurda et al., 2010). Though lacking in concrete proof, such variable systems are often believed to play important roles in pathogenicity via host immune evasion and adaptation. Our data from a recent experimental infection study with Xer1-disrupted Vpma ‘phase-locked*’* mutants (Chopra-Dewasthaly et al., 2008) clearly demonstrated that Xer1 is not a virulence factor of *M. agalactiae* and Vpma phase variation is not necessary for establishing infection though it might critically influence the survival and persistence of the pathogen under natural field conditions (Chopra-Dewasthaly et al., 2012). P40, a cytadhesin, and P48 with macrophage stimulatory activity, are two other lipoproteins, which seem to have important pathogenicity related attributes (Rosati et al., 1999; Fleury et al., 2002). Besides, production of hydrogen peroxide (Khan et al., 2005), biofilm formation (McAuliffe et al., 2006) and identification of genes involved in indirect host cell interactions (Baranowski et al., 2010) are also implicated in *M. agalactiae's* pathogenicity.

In view of the prevailing scenario, we tried to investigate whether *M. agalactiae* has the capacity to enter, survive and exit the eukaryotic host cells in a viable state, as this could explain the chronic, persistent and difficult-to-eradicate nature of its infections in spite of long antibiotic therapies. This phenomenon may also allow it to reach more favorable host niches by crossing the epithelial barrier as cell invasion is often considered a major factor for systemic spread (Cieri et al., 2002; Much et al., 2002). Here, we provide evidence for the first time that *M. agalactiae* is able to invade eukaryotic host cells whereby quantitative results are supported by the qualitative double immunofluorescence assay. Intracellular mycoplasmas were detected not only after in vitro infection but also in vivo in various tissue samples from experimentally infected sheep using immunohistochemistry. Also, by the isolation of mycoplasmas from various internal organs of sheep experimentally infected via the intramammary route we formally demonstrate that *M. agalactiae* has the capability to cross local epithelial barriers and to disseminate to distant body sites. The findings of this study, together with the sophisticated antigenic variation system, could explain the persistence and chronicity of *M. agalactiae* infections.

## Materials and methods

### Mycoplasma growth

*M. agalactiae* pathogenic type strain PG2 (Sirand-Pugnet et al., 2007) was used in this study and was previously isolated from an infected goat in Spain (Fleury et al., 2002). It was grown in Aluotto or SP4 medium supplemented with penicillin, pyruvate, and phenol red as indicator as described before (Chopra-Dewasthaly et al., 2005b). Mycoplasma cultures were grown for 48 h and diluted serially in minimal essential medium (MEM) supplemented with non-essential amino acids and 10% heat inactivated fetal bovine serum (FBS) (Gibco BRL, Life Technologies) prior to infection of cultured mammalian cells. Number of viable mycoplasmas at the time of infection was determined by plating serial dilutions on SP4 plates containing 1% (wt/vol) Difco Noble agar and counting colonies under BMS 74955 stereomicroscope after 4–5 days of incubation at 37 °C.

### Cell culture

HeLa-229 (ATCC CCL-2.1), Bovine endometrium cell line BEND (ATCC CRL-2398) and Buffalo lung fibroblasts (BLF; ATCC IMR-31) were the cell lines used in this study and were purchased from the American Type Culture Collection (ATCC; Manassas, USA) and certified to be free of mycoplasmas. HeLa-229 was maintained in MEM, BLF in McCoy*'*s 5a medium (Sigma) with 10% heat inactivated FBS, and BEND cells in 1:1 mixture of Hams F12 and Eagle*'*s MEM with Earle*'*s BSS (Sigma-Aldrich) as per the instructions of ATCC. Trypsin and PBS were purchased from PAA Laboratories GmbH, Pasching, Austria or Sigma-Aldrich. 1 × 10^4^ cells/well were seeded into Lab-Tek II Chamber Slides (Nunc International, Naperville, IL) for immunofluorescence staining and 5 × 10^4^ cells/well were seeded into 24-well plates (CELLSTAR^®^ Greiner Bio-One GmbH, Germany) for the gentamicin invasion assay 48 h prior to infection to attain confluence. Cell cultures were regularly checked for mycoplasma contamination by culture and PCR.

#### Mycoplasma infection and gentamicin invasion assay

Gentamicin invasion assay was carried out as described before with some modifications (Elsinghorst, 1994; Winner et al., 2000). *M. agalactiae* was grown for about 48 h indicated by metabolic color change before pelleting at 10,000 × *g* at 4 °C for 10 min and resuspending in MEM. The cells were passed through 27-gauge needle for three to four times to disrupt any cell aggregates. Eukaryotic cells were infected with diluted cultures of mycoplasmas at a multiplicity of infection (MOI) of about 10–30 and incubated at 37 °C with 5% CO_2_ for 4, 8, 16 and 24 h. Thereafter, extracellular bacteria were killed by incubation in MEM containing 400 μg/ml of gentamicin for an additional 3 h period. Although a concentration of 50 μg/ml gentamicin is known to be completely inhibitory for *M. agalactiae* growth (Chopra-Dewasthaly et al., 2005b), a higher concentration of 400 μg/ml was used to ensure the reliability of the assay and was experimentally determined to be sufficient to kill 100% of *M. agalactiae* in 3 h duration. After gentamicin treatment, supernatants were checked for the presence of any viable mycoplasmas by plating on SP4 agar. Subsequently, the cells were washed two to three times with PBS and trypsinized before making serial dilutions for plating on SP4 agar to quantify invaded mycoplasmas. Trypsinization did not cause any adverse effects on the mammalian cells as confirmed by the viable cell counts using trypan blue staining. Invasion frequency was calculated as percentage ratio of cfu of intracellular mycoplasmas to cfu of mycoplasmas added initially. Survival and exit of mycoplasmas from the eukaryotic cells was assessed by the same procedure as was described for invasion, except that after 24 h of infection, eukaryotic cells were further incubated in fresh MEM containing 50 μg/ml gentamicin for 8, 16 and 24 h. This lower concentration of gentamicin was checked to be sufficient to prevent the multiplication of mycoplasmas (Chopra-Dewasthaly et al., 2005b). Escape of invaded intracellular mycoplasmas from eukaryotic cells and their reinfection was monitored by incubating the cells in parallel wells under the same conditions in absence of gentamicin. Penicillin (50 μg/ml) was added here as a control antibiotic to prevent general contamination. Cells collected at 8, 16 and 24 h post gentamicin treatment were then serially diluted and plated on SP4 agar. The cfu obtained in presence and in absence of gentamicin were compared with the cfu of invaded mycoplasmas at 24 h pi. Viability of eukaryotic cells was checked regularly by trypan blue staining. All the above experiments were done in duplicates and performed at least thrice under the same conditions.

### Raising M. agalactiae antiserum

M. agalactiae specific antiserum was generated in rabbits as described earlier (Ruhnke and Rosendal, 1994) by subcutaneous inoculation of 10^10^ cfu of *M. agalactiae* type strain PG2 at the Institute of Bacteriology, Mycology and Hygiene, Veterinary Medicine University of Vienna, Austria.

### Double immunofluorescence assay

Double immunofluorescence assay was performed to visually detect *M. agalactiae* inside the eukaryotic cells. The assay was performed as originally described (Heesemann and Laufs, 1985) and previously applied on mycoplasmas (Winner et al., 2000) with minor modifications. Eukaryotic cells were seeded on eight-well Lab-Tek II Chamber Slides (Nalge Nunc International, Naperville, IL) in their respective media and confluent monolayers were infected with mycoplasmas at a MOI of approximately 20 as described above and incubated at 37 °C with 5% CO_2_ for 24 h for optimal invasion. Unbound bacteria were washed away with PBS containing 2% BSA and chamber slides overlaid with 300 μl of 1:200 diluted rabbit anti-*M. agalactiae* serum and gently shaken for 30 min at room temperature. After washing away the excessive antiserum, the cells were covered with fluorescein isothiocyanate (FITC)-labeled goat anti-rabbit immunoglobulin G (IgG) (Invitrogen) as a secondary antibody for 20 min at room temperature with shaking to stain extracellular mycoplasmas. After air-drying, cells were permeabilized for antibody diffusion by successively treating with 50%, 70%, and 96% (vol/vol) ethanol and twice with 100% methanol for 1 min each. The cells were again air-dried prior to treatment with 300 μl of rabbit anti-*M. agalactiae* serum for 30 min before labeling with Texas Red-labeled goat antirabbit IgG (Invitrogen) as secondary antibody for 20 min to stain extracellular and intracellular bacteria. After washing, the nuclei were stained with 300 μl of 1:2000 diluted DAPI (Invitrogen). After final washing, the chambers were removed and cells mounted under a glass coverslip in 1:2 (vol/vol) glycerol-PBS containing 13% (wt/vol) Mowiol (Clariant, Muttenez, Switzerland) and 0.5% (wt/vol) *n*-propyl gallate (Sigma). The cells were examined under OLYMPUS AX 70 epifluorescence microscope with an oil immersion lens (magnification, ×100). Extracellular mycoplasmas were observed as green spots using FITC filter set, whereas the Texas Red filtering revealed both the extracellular and intracellular mycoplasmas as red spots. The two images were superimposed using the Soft Imaging System Cell* from Olympus, Muenster, Germany, whereby intracellular mycoplasmas appeared red and extracellular mycoplasmas appeared yellow due to the overlap of red and green color.

### Systemic spread and reisolation of *M. agalactiae* from different inner organs of experimentally infected sheep

Clinically healthy lactating ewes of the local mountain breed, negative for major sheep pathogens (attested by routine bacteriological and PCR diagnostics) and also confirmed to be seronegative for *M. agalactiae* by two different commercial ELISA kits (Cypress Diagnostics, Langdorp, Belgium and IDEXX Montpellier SAS, Montpellier, France) were infected via the right teat canal as described earlier (Chopra-Dewasthaly et al., 2012). The inoculum, which consisted of 10^9^ bacteria/sheep in 5 ml volume, was prepared as described before (Chopra-Dewasthaly et al., 2012) except that the pellet was resuspended in PBS (Gibco BRL, Life Technologies). The sheep were housed at the Veterinary Medicine University of Vienna, Austria, and infections carried out in accordance with the guidelines of the Austrian law for animal protection with the requisite official approval. Sheep were subjected to regular clinical and serological examination 1 week prior to the intramammary infection and during the entire experimental period of 2 weeks. Presence of bacteria in milk and in mucosa of eye, ear, nose, and genitals was checked regularly by culturing in Aluotto medium as described earlier (Chopra-Dewasthaly et al., 2012). The animals were humanely killed after a fortnight and various organs, such as spleen, lungs, kidneys, udders, heart, brain, uterus, liver and joint tissue, and also various lymph nodes including mandibular, parotid, medial and lateral retropharyngeal, superficial cervical, mediastinal, jejunal, mesenterial, medial iliac, popliteal and supramammary were obtained. A portion of each of these was immediately cultured and the rest of the samples stored at −80 °C in individual sterile vials for subsequent examination. Bacteria from these necropsied specimens were isolated by growing undiluted and diluted tissue samples in Aluotto medium at 37 °C for 7 days. Confirmation of *M. agalactiae* was made by standard biochemical and serological methods as described earlier (Chopra-Dewasthaly et al., 2012) and by performing *M. agalactaie* specific PCR based on 16S rRNA gene (Chavez Gonzalez et al., 1995). Host tissues found positive for *M. agalactiae* were cultured again in SP4 medium (Chopra-Dewasthaly et al., 2005b) for calculating quantitative mycoplasma loads per gram of tissue by excising, weighing and inoculating an estimated small part (about 1 cm^3^) from the frozen −80 °C sample (Chopra-Dewasthaly et al., 2012).

### In vivo detection of *M. agalactiae* by immunohistochemical analysis

Tissue samples collected from necropsied animals were fixed in 10% buffered formalin, alcohol dehydrated, embedded in paraffin wax and stained with haematoxylin-eosin and examined by light microscopy. Additional sections were stained for demonstration of mycoplasmas using a rabbit polyclonal antiserum raised against whole cell antigens of *M. agalactiae* (as described above in Section 2.4). Immunohistochemistry was performed using the HRP polymer method on a Lab Vision-Autostainer (Thermo Fisher Scientific, Fremont, CA). Briefly, the paraffin wax sections (2 μm) were mounted on positively charged glass slides (Superfrost plus; Menzel Glaeser, Braunschweig, Germany) and deparaffinized with Neo Clear^®^ solution (Merck, Darmstadt, Germany) and rehydrated twice in 100% alcohol and once in 96% and 70% alcohol and finally in distilled water. Antigen retrieval was performed by heating the slides in citrate buffer (pH 6.0) in the Lab Vision PT Module (Thermo Fisher Scientific). To reduce nonspecific background staining due to endogenous peroxidase, slides were incubated in Hydrogen Peroxidase Block (Thermo Fisher Scientific) for 5 min, followed by a 10-min incubation in Ultravision Protein Block (Thermo Fisher Scientific). Subsequently, the sections were incubated with the primary antibody (rabbit anti – *M. agalactiae* serum 1:1500) for 30 min at room temperature, followed by the Primary Antibody Enhancer (Thermo Fisher Scientific) for 15 min, and finally with Large Volume HRP Polymer (Thermo Fisher Scientific) for 20 min. Large Volume DAB Plus Substrate System (Thermo Fisher Scientific) was used as chromogen for 5 min. After counterstaining with 1:8 diluted Mayer*'*s Hematoxylin (Thermo Fisher Scientific) for 1 min, the sections were dehydrated in alcohols (70, 96, and 100%) and treated with Neo Clear^®^ (Merck, Darmstadt, Germany) before mounting them in Neomount (Merck, Darmstadt, Germany) for microscopic examination.

### Statistical analysis

Invasion rates are expressed as mean ± standard deviation (SD) of *n* independent values. The significance of differences between means of experiments was calculated by Student*'*s *t* test using GraphPad Prism 5 (Graphpad Software Inc, CA, USA). Differences with *P* *<* 0.05 were considered significant.

## Results

### Entry of *M. agalactiae* into mammalian cells

**Fig. 1:**
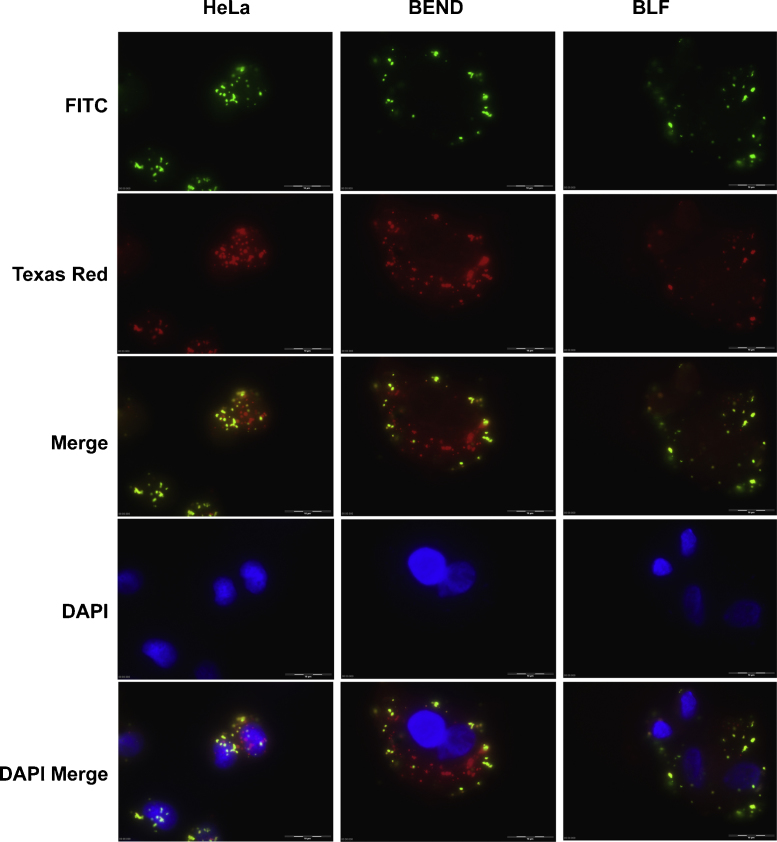
Double immunofluorescence staining showing the invasion of *M. agalactiae* into HeLa-229 and BEND and BLF cells. The five panels correspond to the same area of the infected monolayer. FITC fluorescence showing extracellular mycoplasmas stained green, Texas Red fluorescence showing extracellular and intracellular mycoplasmas stained red, and DAPI fluorescence showing cell nuclei stained blue. Merged images indicating the localization of extracellular (yellow) and intracellular (red) mycoplasmas. Bars, 10 μm.

**Fig. 2:**
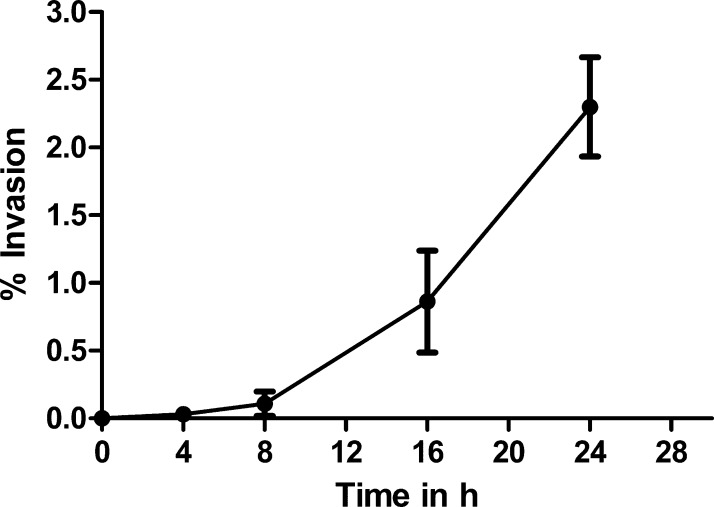
Invasion of *M. agalactiae* type strain PG2 into cultured HeLa-229 cells at different times post infection. The percentage invasion was calculated by dividing the cfu value obtained after gentamicin treatment with the cfu value of total mycoplasmas added for infection and multiplied by 100. The data represent mean (±SD) of four independent experiments performed in duplicate.

The ability of *M. agalactiae* to invade mammalian cells was investigated by infecting three different mammalian cell lines with the pathogenic type strain PG2. Apart from HeLa, which is a standard epithelial cell line used in many mycoplasma invasion studies (Andreev et al., 1995; Winner et al., 2000; Yavlovich et al., 2004; Marques et al., 2010; Fürnkranz et al., 2013; Hopfe et al., 2013), gentamicin invasion assays were also performed on cultured ruminant cells, namely BEND and BLF. PG2 cells were incubated with these mammalian cells at an MOI of 10–30 for 4, 8, 16 and 24 h before subjecting them to gentamicin treatment to kill extracellular bacteria and then directly plated onto SP4 agar to enumerate viable intracellular bacteria by cfu counts. Each cfu represents an infected eukaryotic cell and might be actually corresponding to more than one mycoplasma cell residing in the same eukaryotic host cell. This implies that the actual invasion frequency might be higher than the calculated value as most infected cells were observed to harbor multiple mycoplasma cells in them (Fig. 1). The invasion frequency was expressed as percentage ratio of number of recovered intracellular bacteria to the number of bacteria used for the initial infection. For HeLa cells an increase in invasion frequencies was observed with increasing infection times (Fig. 2). The invasion at 4 h post infection was quite low (0.032% ± 0.01) and thereafter increased exponentially with highest rates witnessed at 24 h post infection (2.28% ± 0.19). Such long infection periods and/or comparable invasion frequencies have earlier been reported for many bacterial pathogens, including mycoplasmas, which were predominantly known to be extracellular but were subsequently shown to have alternative intracellular lifestyle (Martin and Mohr, 2000; Dusanic et al., 2009; Marques et al., 2010; Buim et al., 2011; Hopfe et al., 2013; Lamberti et al., 2013). The percentage invasion frequency differed slightly for the three cell types. At 24 h post infection, the invasion frequency for BEND cells (2.14% ± 0.8) was comparable to HeLa but BLF cells demonstrated a relatively lower frequency of 1.14 ± 0.5. It was checked that incubation of mycoplasmas in MEM medium alone for this time period of 24 h yielded negligible or no increase in their numbers.

Invasion of *M. agalactiae* into non-phagocytic cells was further confirmed by double immunofluorescence staining as used for other mycoplasmas (Winner et al., 2000) with some modifications as mentioned above under Section 2.6. Since the gentamicin invasion assay had indicated optimal invasion rates at 24 h post infection, this time point was selected for fluorescence staining to enable proper readouts. The results clearly demonstrate the intracellular status of *M. agalactiae* as seen in Fig. 1, which shows five micrographs, each corresponding to the same area of infected HeLa, BEND and BLF cells. Extracellular mycoplasmas were observed as green spots using FITC filter set, whereas the Texas Red filtering revealed both the extracellular and intracellular mycoplasmas as red spots. The two images were superimposed and intracellular mycoplasmas appeared red. As controls, uninfected eukaryotic monolayers were also stained to rule out the possibility of any previous contamination with mycoplasmas and/or any cross-reactivity of antibodies. In agreement with the gentamicin invasion assay results, BLF cells showed comparatively fewer intracellular mycoplasmas via double immunofluorescence as compared to BEND and HeLa cells (Fig. 1).

### Fate of *M. agalactiae* after entry into the eukaryotic cells

**Fig. 3:**
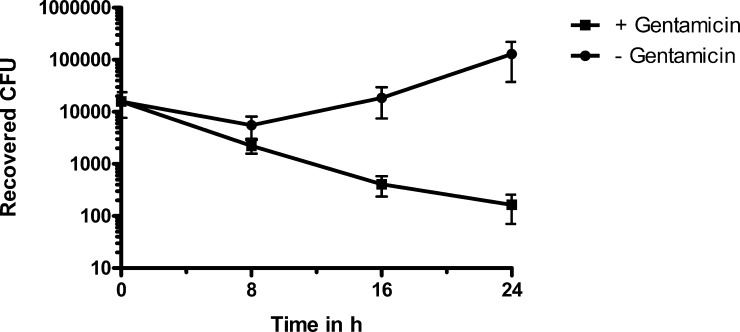
Fate of *M. agalactiae* after entry into the eukaryotic cells. HeLa-229 cells were incubated with *M. agalactiae* type strain PG2 for 24 h followed by gentamicin treatment for 3 h at 37 °C. Cells were washed and incubated with fresh MEM with (black squares) or without (black circles) gentamicin for additional 8, 16 and 24 h, and then trypsinized and plated to enumerate the viable mycoplasmas. Mean values ± SD from three independent experiments performed in duplicate are indicated.

Cell invasion and subsequent intracellular survival, intermittent or prolonged, is an important strategy of many successful pathogens to evade the host immune response. Not just the entry but also the exit from the host cell is a critical step for an intracellular pathogen (Hybiske and Stephens, 2007, 2008; Friedrich et al., 2012; Lamberti et al., 2013). Therefore, fate of *M. agalactiae* was evaluated after its entry into HeLa cells. This was done by infecting HeLa cells with *M. agalactiae* for 24 h and further treating them with gentamicin to kill extracellular bacteria. This was followed by additional incubations for 8, 16, or 24 h in absence, as well as in presence of gentamicin at a concentration of 50 μg/ml. Viable intracellular bacteria were enumerated at these different times by cfu counts as described in Section 2.3. The results, as shown in Fig. 3, are very different for the cells incubated in presence of gentamicin as compared to those that were incubated in absence of the same. In presence of gentamicin, a continuous decrease in the cfu counts is observed until the last tested time point of 24 h of incubation. Compared to this, in absence of gentamicin the cfu count is not going down and rather shows an increase by 24 h of incubation. Two possibilities could explain the latter results whereby the bacteria are either replicating extracellularly or intracellularly. But intracellular multiplication is ruled out as parallel incubations in gentamicin show a continuous decrease in the cfu counts. Taken together, these results imply that intracellular *M. agalactiae* is released in a viable state into the surrounding medium and possibly multiplies extracellularly to reinfect new host cells, as observed in case of incubation in the absence of gentamicin. Since we did not see any cell death in parallel uninfected wells during 24 h infection, the exit of *M. agalactiae* is likely not due to cell lysis caused by culture overgrowth. As a control, presence of gentamicin was checked to have no adverse effects on the viability of eukaryotic cells.

### In vivo systemic dissemination of *M. agalactiae* to distant body sites during experimental intramammary sheep infection

**Fig. 4:**
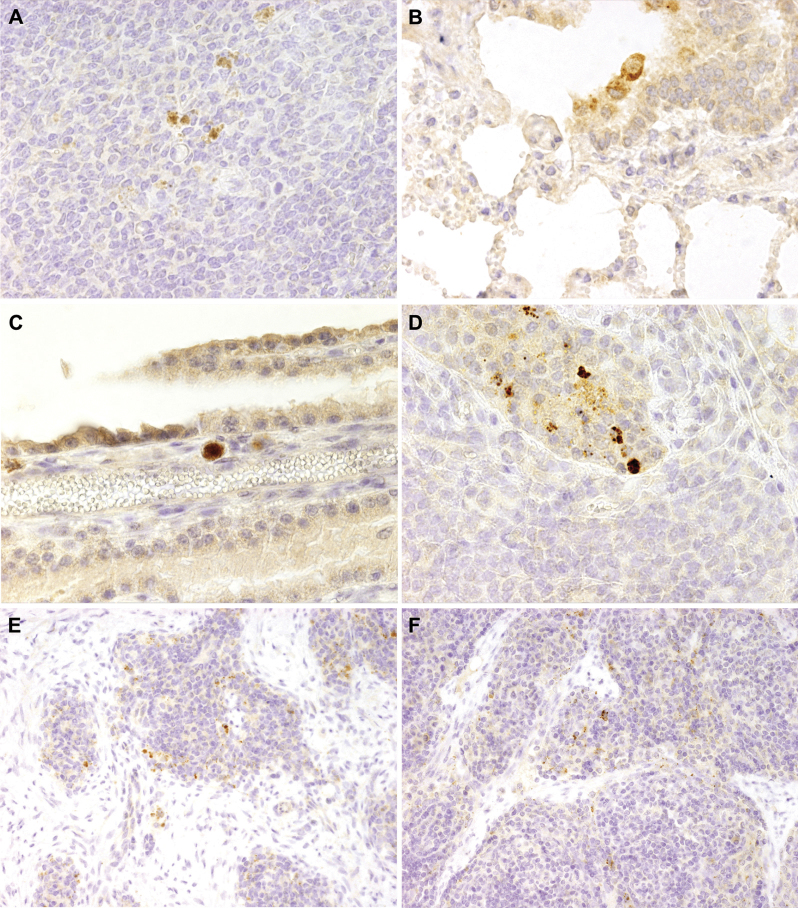
Immunohistochemical detection of *M. agalactiae* in samples obtained from sheep experimentally infected via the intramammary route and necropsied at Day 16 p.i. *M. agalactiae* specific signals are observed in spleen (A), lung (B), choroid plexus of brain (C), udder (D) and, left (E) and right (F) popliteal lymph nodes respectively, using anti-*M. agalactiae* specific rabbit polyclonal antibodies. (A)–(D), ×40; (E) and (F), ×20.

*M. agalactiae* causes chronic and persistent infections in small ruminants and the infected animals continue to shed the bacteria for several months, sometimes for several years (Bergonier et al., 1997; Gomez-Martin et al., 2013). Cell invasion is believed to play a major role in the systemic spreading of many pathogens, including mycoplasmas (Cieri et al., 2002; Much et al., 2002). Apart from a report describing the isolation of *M. agalactiae* from naturally infected goats (Gomez-Martin et al., 2012) there are hardly any such reports based on experimental infections of lactating sheep. However, sporadic isolation of *M. agalactiae* (P89) was observed in spleen and lungs of very few lambs experimentally infected via the conjunctival route (Sanchis et al., 1998). We wanted to formally address the ability of *M. agalactiae* to cross the epithelial barrier of the udder and disseminate throughout the body by demonstrating its isolation from various internal organs during an experimental intramammary sheep infection*.* Routine milk and mucosal swab samples, as well as the necropsied tissue and lymph node samples were checked for the reisolation of *M. agalactiae* via culture and PCR methods as described earlier (Chavez Gonzalez et al., 1995; Chopra-Dewasthaly et al., 2012). Data demonstrated the presence of *M. agalactaie* in various internal organs collected from infected animals (Table 1). Apart from the expected organs such as udders and lymph nodes, *M. agalactiae* was also detected in liver, lungs, uterus, kidneys, heart, brain, and carpal and knee joint tissues (Fig. 4). The results clearly illustrate the successful dissemination and bilateral spreading of the pathogen from the single site of infection, that is, the right teat canal, to several distant body sites, including heart and brain (see Table 1). Quantitative mycoplasma loads in organs, such as uterus, udder, popliteal lymph nodes and lungs varied between 10^3^ and 10^5^ cfu/g of tissue (Table 2). All samples found positive in *M. agalactiae* specific PCR did not show mycoplasma loads during quantitative analysis. This might be due to the already low mycoplasma count in these samples that die further during the sensitive freeze/thaw cycles needed for cfu enumeration. Overall, the results show for the first time the systemic spread of *M. agalactiae* to new anatomic sites during experimental intramammary infection of sheep.

### Immunohistochemical demonstration of *M. agalactiae's* cell invasiveness during an in vivo infection

Detection of *M. agalactiae* in ovine mastitis by immunohistochemistry has not been reported so far. Various tissue samples obtained at necropsy were examined by immunohistochemistry using PG2 specific polyclonal antiserum to determine the presence of *M. agalactiae* in tissue/organs of infected animals. As illustrated in Fig. 4D, *M. agalactiae* antigens were clearly visible in the cytoplasm of mammary duct epithelium. Not only in the expected udder tissue, but also for the first time, *M. agalactae* was detected in the distant internal organs, such as the lungs (Fig. 4B), brain (Fig. 4C) and spleen (Fig. 4A) of experimentally infected sheep using immunohistochemistry technique. Interestingly, immunohistochemical staining of the lung (Fig. 4B) and brain tissue (Fig. 4C) demonstrated the presence of *M. agalactiae* in the cytoplasm of macrophages. Except for spleen, these results are in agreement with bacterial culture and PCR results, reconfirming *M. agalactiae's* capability to spread into anatomically distant body parts during experimental infections.

## Discussion

Mycoplasmas are generally considered extracellular pathogens although in the last decades there have been quite a few reports providing sufficient evidence for some of these species to be capable of invading eukaryotic host cells (Fürnkranz et al., 2013). However, for *M. agalactiae*, which is well known for its chronic and persistent infections, cell invasion, and for that matter even a precise or direct account of its cytadherence capability, considering adherence to be a prerequisite for invasion, is yet to come. This is in accordance with the fact that it fails to show the more convenient phenotypes of hemadsorption and terminal tip structure unlike some other invasive *Mycoplasma* spp. (Rosengarten et al., 2000; Winner et al., 2000; Rottem, 2003). The data presented in this study provides the first evidence about the cell invasion capability of *M. agalactiae* pathogenic type strain PG2. Presence of *M. agalactiae* was demonstrated in the standard HeLa cells, as well as in two different ruminant cell lines, namely BEND and BLF, using the quantitative gentamicin invasion assay and the qualitative visual method of double immunofluorescence staining. Though the highest invasion frequency of *M. agalactiae* is calculated to be around 2.3% at 24 h post infection, it is comparable and even better than the reported invasion frequencies of some other pathogens, especially mycoplasmas (Martin and Mohr, 2000; Cieri et al., 2002; Dusanic et al., 2009; Marques et al., 2010; Buzinhani et al., 2011)

Furthermore, apart from the in vitro proof of *M. agalactiae* cell invasion, an in vivo experimental infection study was also performed, whereby the pathogen was demonstrated to be capable of crossing the epithelial barrier of the infected right udder and disseminating throughout the body as confirmed by its reisolation from various internal organs. Cell invasion capacity of pathogens is often believed to play a significant role in their systemic spread (Winner et al., 2000; Cieri et al., 2002; Much et al., 2002). Whether *M. agalactiae's* modest cell invasiveness actually correlates with the observed systemic infection needs to be formally demonstrated. But the fact that *M. agalactiae* is capable of entering and escaping cultured eukaryotic cells and its bilateral presence in many different host internal organs away from the site of initial in vivo infection can be well explained, and perhaps indirectly correlated, with its ability to invade local epithelial cells and/or to spread to draining lymph nodes before becoming systemic. This is supported by the immunohistochemical detection of *M. agalacatiae* in the cytoplasm of mammary duct epithelium (Fig. 4D) and macrophages present in different organs (Fig. 4B and C). To our knowledge this is the first study which has formally proved, not only the entry of *M. agalactiae* into host cells, but has also shown using immunohistochemistry staining, the inter- and intra-cellular residence of this pathogen in distant internal organs, such as lungs (Fig. 4B), spleen (Fig. 4A), brain (Fig. 4C), and in the knee and carpal joints (Fig. 4E and F) of sheep experimentally infected via the right teat canal. Especially interesting is the immunohistochemistry result where *M. agalactiae* is seen in the cytoplasm of macrophages in the lungs (Fig. 4B) and choroid plexus tissue of the brain (Fig. 4C). This implies that either the mycoplasma is capable of actively invading these phagocytic cells, or it has partially or fully survived the macrophage phagocytosis and subsequent immune clearance at the time of necropsy. Both possibilities are equally intriguing and would provide a fresh insight into the pathogenicity mechanisms of *M. agalactiae.*

An important finding of the present study is that the internalized *M. agalactiae* is released into the extracellular media in a perfectly viable state, though it is difficult to conclude anything about its intracellular replication under the given experimental conditions. After exiting the host cells, mycoplasmas tend to multiply and reinfect new host cells. This in vitro scenario could very well reflect the sequence of events occurring in vivo during the experimental intramammary infection, where the bacteria is speculated to have translocated through various host cell layers by cell invasion, exit and reinfection, thereby leading to the systemic spread of infection.

As for most other invasive mycoplasmas, the precise mechanism employed by *M. agalactiae* to enter non-phagocytic host cells is unknown. It is likely that surface proteins facilitating adhesion will have an effect on invasion, though adherence is not sufficient to trigger invasion events. *M. agalactiae* lacks a dedicated terminal tip structure where adhesion related molecules are localized for efficient cytadherence of some important mycoplasma pathogens (Rottem, 2003). Except for P40 (Fleury et al., 2002), no other cytadhesin has been identified in *M. agalactiae*, although the Vpma family of variable surface lipoproteins were shown to contain adhesion epitopes found in the homologous Vsp proteins of *M. bovis* (Glew et al., 2002), a close phylogenetic relative of *M. agalactiae* (Askaa and Ernø, 1976). Further investigations into the detailed invasion mechanisms of *M. agalactiae*, including the identification of mycoplasma adhesins and invasins and also eukaryotic host receptors will add to our understanding of this important ruminant pathogen.

It is thus clear that like many other bacterial pathogens, the division between intracellular and extracellular mycoplasmas is becoming increasingly blurred as more and more pathogens originally believed to be extracellular are now shown to have alternative intracellular lifestyles (Bower et al., 2005; Fürnkranz et al., 2013; Lamberti et al., 2013). This alternative capacity of *M. agalactiae*, even if it is exhibited by a very small subpopulation of total infected cells, might provide the pathogen with a gross advantage at the population level to hide from antibiotics and host immune responses and to navigate through the host body to reestablish infection in new host niches, thus causing persistent infections that are difficult to eradicate.

## Figures and Tables

**Table 1:** Qualitative bacteriological examination of lymph nodes and organs from sheep inoculated by the intramammary route with 10^9^ viable cfu of *M. agalactiae* type strain (PG2) and necropsied at Day 16 p.i.

Organs/Tissue samples	PCR^a^	Culture^b^	Immunohistochemistry^c^
Supramammary LN^d^ right	+	+	+
Parotid LN right	+	+	ND^e^
Parotid LN left	+	+	ND
Popliteal LN right	+	+	+
Popliteal LN left	+	+	+
Liver	+	+	−
Udder right	+	+	+
Udder left	+	+	−
Lung left	+	+	+
Lung right	+	+	+
Kidney right	+	+	ND
Spleen	−	−	+
Heart	+	+	−
Uterus (fallopian tube) right	+	+	−
Uterus left	+	+	−
Capsule (carpal joint) left	+	+	ND
Capsule right	+	+	ND
Brain	+	+	+
Synovial fluid (knee joint) right	+	+	ND

a *M. agalactiae* 16S rRNA specific PCR results.

b *M. agalactiae* colonies obtained on Aluotto agar by plating the broth subcultures of various tissue and lymph node (LN) samples.

c IHC: immunohistochemical analysis of selected *M. agalactiae* cultures.

d LN: lymph node.

e ND: not done.

**Table 2:** Quantitative bacterial examination of lymph nodes and organs from sheep inoculated by the intramammary route with 10^9^ viable cfu of *M. agalactiae* type strain PG2 and necropsied at Day 16 p.i.

Organ/lymph node	Mycoplasma load^a^
Supramammary LN^b^ Right	1.8 × 10^4^
Parotid LN left	5 × 10^3^
Parotid LN right	5 × 10^3^
Uterus (fallopian tube) right	2.46 × 10^6^
Popliteal LN left	5 × 10^3^
Popliteal LN right	5 × 10^3^
Udder right	4.79 × 10^6^

a Mycoplasma load is represented as cfu/g of tissue/organ/lymph node.

b LN (lymph node).
